# The Helicopter Transports of Patients Critically ill with COVID-19

**DOI:** 10.1177/23779608221094108

**Published:** 2022-04-21

**Authors:** Jennifer Ejderlöf, Åsa Engström

**Affiliations:** 1 Intensive Care Unit, Gällivare Hospital, Gällivare, Sweden; 2 Department of Health, Education and Technology, Division of Nursing and Medical Technology, 91874 Lulea University of Technology, Luleå, Sweden

**Keywords:** COVID-19, helicopter transport, intensive care, specialist nurse

## Abstract

**Objective:**

The aim of our study was to describe specialist nurses’ experiences with the helicopter transport of patients critically ill with COVID-19.

**Method:**

Our study followed a descriptive qualitative design anchored in the naturalistic paradigm and was based on qualitative content analysis. The study followed the consolidated criteria for reporting qualitative research.

**Results:**

Six semi-structured interviews were conducted with specialist nurses who have cared for patients critically ill with COVID-19 during helicopter transport. The analysis of the interviews resulted in three themes—designing new routines before transport, working under new conditions and post-transport and reflections—with 11 categories. The goal of the intensive care transport of patients with COVID-19 was twofold: to prevent the spread of infection by using personal protective equipment and to prevent the contamination of the helicopter.

**Conclusion:**

For the nurses, working in personal protective equipment created a feeling of distance from patients that compromised patient–nurse intimacy. Our results suggest that ensuring the sufficiency of equipment required in the event of major accidents and pandemics is critical.

## Introduction

When patients urgently need transport that involves travelling long distances and/or accessing remote areas, ambulance helicopters are often used (Diaz et al., 2005). The aim of our study was to describe specialist nurses’ experiences with the helicopter transport of patients critically ill with COVID-19.

## Literature Review

To transport patients requiring intensive care, helicopters and their care staff need to have the appropriate equipment and competence. As for competence, experience in intensive care and transport is considered to be pivotal in aviation medicine (Frost et al., 2019; Martin & Kumar, 2020; Topley et al., 2003), as is knowledge of airway management and general emergency care (Martin & Kumar, 2020). For those reasons, planning is an important part of preparing to deliver care (Frost et al., 2019; Senften & Engström, 2015; Topley et al., 2003) and to establish plans for various situations that may arise (Frost et al., 2019). Such planning is facilitated by controlled conditions and in light of available information about patients prior to departure (Topley et al., 2003).

Due to the specific environment within helicopters, nurses also have to be competent in adapting how they assess patients (Topley et al., 2003). Spatially, the small surface area within helicopters and low ceiling height present particular challenges (Frost et al., 2019; Martin & Kumar, 2020; Senften & Engström, 2015). During transport, loud helicopter noise also typically prevents nurses from being able to hear patients’ breathing (Senften & Engström, 2015; Topley et al., 2003), which requires them to assess patients primarily via visual observation (Pugh, 2002; Senften & Engström, 2015; Topley et al., 2003).

As another form of competence, the collaboration of the care team in helicopter transport is vital as well (Frost et al., 2019; Topley et al., 2003). Collaboration amongst care staff has a calming, positive effect on patients (Sandström et al., 2017), which can be a major benefit for patients who have described helicopter transport as a frightening way to get to the hospital (Sandström et al., 2017). Common diagnoses of critically ill patients transported by helicopter are multiple trauma, acute myocardial infarction, unstable coronary heart disease, burns and infection (Sveriges Kommuner och Landsting, 2012). Added to that, since spring 2020, patients critically ill with COVID-19 have been transported via helicopter to specific COVID-19 intensive care units (ICUs) at long distances (Bredmose et al., 2020). In northern Sweden and elsewhere, nurses who have worked in helicopters have had to rapidly transition in preparation to receive patients with COVID-19 by designing new work routines. According to the Swedish Nurses’ Association (Svensk Sjuksköterskeförening, 2020), nurses specialising in intensive care need to be able to care for critically ill patients during transport both within and between hospitals, as well as to initiate, measure and improve the development of the intensive care that they provide.

Despite such trends and needs, research on intensive care transport via helicopter and on the transport of intensive care patients with COVID-19 has been limited. The research that has been conducted has sought to capture the general experiences of medical professionals with the helicopter transport of critically ill or injured patients. As a consequence, research specifically addressing specialist nurses’ experiences with the helicopter transport of patients critically ill with COVID-19 has been quite limited. However, such research can contribute to useful knowledge about similar forms of transport and situations during ongoing and upcoming pandemics. For those reasons, the aim of our study was to describe specialist nurses’ experiences with the helicopter transport of patients critically ill with COVID-19.

## Methods

To describe specialist nurses’ experiences with the helicopter transport of patients critically ill with COVID-19, our study followed a descriptive qualitative design (Polit & Beck, 2020) anchored in the naturalistic paradigm. In particular, it involved qualitative content analysis, which aims to provide knowledge and understanding of the phenomenon being studied (Graneheim & Lundman, 2004) and is thought to be useful for exploring peoples’ experiences with specific phenomena. In conducting the study, we additionally followed the consolidated criteria for reporting qualitative research (COREQ; Tong et al., 2007).

### Setting

In Sweden, ambulance helicopters are active in nine regions, and all can perform both primary and secondary missions. Whereas primary missions involve retrieving patients directly from the scene of injury or illness, secondary missions involve transporting patients who need intensive care between different care facilities. In the helicopter operation in northern Sweden that we studied, the helicopter is used to transport patients between care facilities within and outside the particular county that the operation serves. The operation is staffed around the clock with anaesthesiologists and specialist nurses possessing expertise in intensive care, anaesthesia and/or ambulance care. The 10 nurses who work in the helicopter spend half of their time providing helicopter-based care and the other half performing other operations in the region. In 2019 and 2020, the operation completed 406 and 341 transports, respectively, and in 2021, as of 31 March, had performed 42 transports of patients with COVID-19 in need of intensive care.

### Ethical Considerations

The head of the helicopter operation granted us permission to conduct our study, after which the Swedish Ethical Review Authority approved the research (No. 2020-02805). All participants agreed to participate by providing their informed, written consent, and before their interviews, all participants received oral and written information about the nature of the study. They were also assured that their participation was voluntary and that they could withdraw from the study at any time for any or no reason. Once each interview began, the first author confirmed that the participant understood the study's aim and guaranteed them confidentiality in the presentation of the findings.

### Data Collection

To participate in our study, nurses had to have a specialist degree in intensive nursing care or anaesthesia nursing care and be experienced with providing intensive care to patients with COVID-19 during helicopter transport in northern Sweden.

We collected data in spring 2021 using semi-structured interviews following an interview guide with both open-ended and follow-up questions. The open-ended questions focused on the participants’ experiences with caring for patients critically ill with COVID-19 during helicopter transport and how those experiences have influenced their everyday lives. In turn, general follow-up questions included “Would you please give an example?” and “How did you feel then?” The interviews, lasting 25–50 min, were conducted over the phone, recorded and transcribed verbatim by the first author.

### Participants

Six specialist nurses, all of whom fulfilled the inclusion criteria, were informed about the study and provided their written consent to participate. At the time of the interviews, each had worked for the helicopter operation in northern Sweden for 1–19 years—in fact, all worked for the same agency—and had experience with providing intensive care to patients with COVID-19 during helicopter transport in the region. Two participants were women, four were men, and their median age was 44.5 years (range: 37–47 years). Five participants had a specialist nursing degree in intensive care, whereas the other had a specialist degree in anaesthesia care.

### Data Analysis

Both authors read the transcribed interviews several times in order to gain a sense of the content as a whole. During analysis, we discussed the findings in seminars together with other researchers and students; by moving back and forth between the text and the results of the analysis, we gradually refined the findings together with them. Once we had identified similarities and differences in the data between the various categories, we subsumed the final 11 categories into three themes that reflected threads of meaning in the categories based on the latent content of the text. Each author then analysed the findings independently before collaborating with the other author to reach a consensus. Last, we read the transcripts once again to confirm and validate the three themes and the 11 categories (cf. Graneheim & Lundman, 2004).

## Results

The results of our study are presented according to three themes with 11 categories (see Table 1) and illustrated with quotations from the participants.

**Table 1.** Overview of the Three Themes and 11 Categories.

Theme	Category
Designing new routines for transport	*Fearing working with patients with COVID-19* *Designing new routines together* *Preparing and planning for the transport of patients with COVID-19* *Preparing patients at the ICU* *Securing patients before transport*
Working under new conditions	*Working and communicating in PPE* *Assessing and treating patients with a novel virus* *Not having relatives involved in the patient's care*
Post-transport and reflections	*Decontaminating the helicopter post-transport* *Impacting one's private life* *Gaining new experiences after working with patients with COVID-19*

### Designing new Routines for Transport

#### Fearing working with patients with COVID-19

Participants reported feeling unprepared for the COVID-19 pandemic; they had thought that the virus would not affect them, and when it did, they expected the worst. High death rates in Italy, newspaper articles about dead nurses and physicians and widespread uncertainty about the severity of the virus caused them to feel fearful and anxious:

From the very beginning I didn't realise that it would be as big a thing as it became and so extensive and affect the whole world. (Participant 2)

Even so, the participants viewed performing the work as their duty because no one else could. They thought that they risked death if they did not perform all tasks flawlessly and worried about how many of their colleagues would need respiratory care or die as a result of COVID-19:

I wondered how many of us would end up in a respirator, how many of us would die and which of us would die. (Participant 6)

#### Designing new routines together

Participants reported that because the helicopter transport of patients with COVID-19 was as novel as the coronavirus itself, the operation's staff had to develop and design new routines together and communicate them to all parties involved. As a result, participants had to not only keep abreast of current information at all times but also continuously process a great deal of fragmented information, which only prolonged the time before all staff members’ work followed the same routines and guidelines. In the meantime, they experimented with strategies to identify ones that would succeed and developed ways of working based on their continually developing foundation of knowledge:

There have been a lot of new routines for doing things and different ways of dealing with [the new routines]. … I’ve had to stay up to date with new things all of the time. (Participant 2)

We are used to, because we have this expectation when we work pre-hospital that anything, anything can happen, and all you can do is try to solve [the problem at hand]. But [COVID-19] was something that we’d never experienced before. (Participant 1)

#### Preparing and planning for the transport of patients with COVID-19

The participants described preparing themselves for transporting patients with COVID-19 by thinking through assignments and, for example, eating and using the toilet before putting on their personal protective equipment (PPE). Using PPE to the extent that the pandemic required was a new moment for participants, and together with physicians, they planned procedures and reviewed what equipment and medicines would be needed during transport. The participants had to verify that protective suits were indeed available, that the filters for protective masks and the stretcher functioned properly, that the correct equipment was on board and that the oxygen supply would be sufficient:

Everything has become more complicated to perform because it has to be planned well, with protective equipment, and we have to, for example, prep the helicopter and ourselves. (Participant 2)

The participants explained that activating the SOS alarm alerted the road ambulance to meet them upon their arrival at the hospital. However, before the participants donned their PPE and went into the ICU, they needed a plan. Although their interviews showcased how ways of preparing differed from person to person, all participants indicated that the sooner the transport order was received, the better prepared they could become. Indeed, the transports were described as having largely succeeded precisely because of good planning and preparation:

You simply wanted a plan and to plan things carefully. (Participant 1)

#### Preparing patients at the ICU

Participants described how the ICU staff prepared patients prior to transport. Working with them, the participants moved patients onto stretchers, equipped them with respirators and administered medication via syringe pumps. Because patients needed to exhibit stable circulation and respiration before being transported, they were generally intubated with a closed suction system and examined by X-ray to determine the location for endotracheal tubes and central catheters. To reduce the risk of infection and to protect against the cold, the patients were typically covered in bubble wrap, and only their endotracheal tubes and central catheters protruded from the plastic:

We learned to wrap them in plastic like they were in cocoons. (Participant 4)

#### Securing patients before transport

Participants described the riskiness of moving patients because, among other complications, hoses could become stuck and endotracheal tubes could become detached. For many, the worst-case scenario was having a patient's circulation become unstable or having a tube get clogged while inside the helicopter. Participants especially feared problems in the air because it was difficult to access the patients while they were wrapped in plastic; therefore, they described double-checking all aspects and equipment prior to transport. By controlling entrances, the location of the endotracheal tubes and the tubing itself, they were able to prevent problems from occurring:

As long as [the patients] were on a respirator, they were quiet because of all of the filters that they have. But there wasn't a lot of COVID blowing around into the cabin because there was a filter. But because leaks were possible … you had to be more careful that all of the equipment was set up as it should be. (Participant 5)

### Working Under new Conditions

#### Working and communicating in PPE

Participants reported learning a great deal about PPE in connection with the COVID-19 pandemic, especially after being provided with the equipment and completing training about its use. Although some participants initially feared PPE shortages, others did not, nor did they worry about getting sick. On the contrary, they believed that anxiety over COVID-19 was overblown and that PPE, though plentiful, was overused. At the same time, those same participants also described always being able to obtain protective masks as a staple of work in their field. Regardless of their views on PPE, the participants described learning how to use the equipment as an intensive process. Initially, they taped their sleeves and trouser legs to prevent viral infection under their protective clothing. Once participants understood how the PPE worked and that it could indeed protect them from COVID-19, their anxiety waned.

Participants found working in PPE to be difficult, especially over prolonged periods. The plastic aprons in particular were perceived as being sticky. Working in PPE also caused dehydration, overheating and physical fatigue, and participants reported that the heat created by the gear could become overwhelming and thus demanded extraordinary concentration when caring for patients. During the summer, the helicopter's large, unopened windows only intensified the heat in the cabin, and in response, the participants prepared by wearing thin hospital clothes under their PPE. Working in PPE during the winter, meanwhile, was described as causing undue cold, for participants wore only underwear underneath for fear of contaminating their normal work clothes. Thus dressed for indoor temperatures, they often became cold while walking to and from the helicopter and while working inside it. As temperatures fluctuated between hot and cold, their visors would fog up, and they could not see where they were going.

The participants described working in protective masks for 2–3 h at a time, during which could not drink water or take deep breaths. They added that the masks resisted normal breathing and required additional effort when inhaling, as well as caused pain on the bridge of the nose. They also described the experience of wearing PPE as being surreal, especially while entering facilities through plastic curtains and pandemic-ready hospital doors into small operating rooms where patients with COVID-19 lay cross-legged.

Participants also reported feeling distanced from patients due to the PPE. They described usually communicating with physicians and pilots via the microphone in their flight helmets; however, during the initial phase of the pandemic, they could not wear helmets due to the PPE, and communication with pilots in the sealed cockpit thus became problematic, if not impossible. To be able to transport patients with COVID-19, they accepted that they would lack full communication with pilots and would therefore not meet a minimal requirement of aviation safety:

It's already difficult enough to talk with a face mask on, but it's even more difficult to communicate while wearing a mask in a helicopter. … So we knew that we would have a hard time talking to each other. … We had to talk through a microphone, but you can't put the microphone in your mouth, or else there would be problems. We knew that, but again, we simply planned ahead, and then it wasn't as much of a problem. (Participant 1)

Regarding patients, the participants reported being able to communicate with ones who were awake but that talking to patients in PPE was generally difficult. They had to communicate with physicians by using body language (e.g. eye contact and hand gestures) and/or by writing on paper or a board when they could not hear each other due to the helicopter's background noise and the known difficulty of communicating through respiratory PPE. Communication nevertheless succeeded because the participants had planned for the difficulty of communicating. They stated that safety was never compromised and that it was possible to shout various commands during dangerous moments such as take-off and landing. In time, they received protective masks with built-in microphones that allowed them to communicate with patients, each other and the pilots:

It felt … like you were more distanced from the patient because you were so trapped in the PPE, but I think that we got used to that. (Participant 6)

#### Assessing and treating patients with a novel virus

Participants described how patients with COVID-19 experienced different sorts of respiratory failure and that most of the one whom they transported were young and sometimes quite ill and/or heavy to lift.

The participants also feared the virus's high contagiousness and thus worked with that fear in mind. Although it was initially unclear how the virus spread, they quickly realised that the virus was not as contagious as they had expected and that there was less risk of being infected at work than at a grocery store, for instance. Initially, to reduce the spread of infection, patients were intubated during transport, and the nurses had created closed respiratory circuits and used transport respirators with extra filters. If they transported patients who coughed and were not intubated, then they knew that the virus was spreading in the cabin.

Participants described great media interest in the helicopter transports and how strangers would watch and take photos of them as they loaded patients into helicopters. They were therefore careful to conceal patients on the stretcher from view:

We were careful to conceal who we had on the stretcher because there were so many people watching us bring them in. There was a lot of media interest around it, lots of people who stood with cameras. So … it's like a combination of protecting purely the patient but also protecting us. (Participant 3)

Participants reported deeply anaesthetising patients with COVID-19 during transport in order to steel them against the activity occurring in the helicopter. They also administered muscle relaxers to patients to prevent coughing and breathing against the respirators. All patients had inotropic drugs connected to syringe pumps that could be quickly activated if necessary:

You didn't want them to cough. It was very muscle-relaxing, the drug that we gave them. We made sure that they were heavily anaesthetised so that they wouldn't chew on the tubes. … There were pressure-increasing drugs and a lot of sedation before transport to avoid any … unnecessary situations. So you quickly made sure that the hoses wouldn't slip apart or connections and so on. … There was a feeling that you … had to be very careful. (Participant 3)

Participants added that they maintained life-sustaining primary care and ensured that all patients survived transport. However, they could not control body angles or pressure ulcers, and patients could become quite slimy during transport due to helicopter vibrations that increased the release of mucus and impaired their breathing. At the same time, ventilators issued excessive backpressure and needed to be sucked clean into the endotracheal tubes. Other participants described not hearing alarms from the monitoring equipment during transport and the impossibility of using a stethoscope, which required using visual observation to gauge abdominal movements. Even so, observing abdominal movements when patients were covered in bubble wrap often proved to be difficult. Although participants found that patients with COVID-19 were better ventilated in the abdominal position, that position was difficult to achieve in the helicopter, where most patients had to be supine and where turning patients over was impossible:

It was difficult to transport [patients] in the abdominal position, but they were much better ventilated if they were in that position. (Participant 4)

#### Not having relatives involved in the patient's care

Participants described forming relationships with patients via patients’ relatives. Relatives told them about the patients and their backgrounds, which made the participants emotional. Due to restrictions on hospital visits during the pandemic's early phases, participants did not have contact with patients’ relatives as they had before. During transport, relatives sometimes watched from a distance, and some later asked how the transport had proceeded. Participants thus believed that patients’ relatives felt excluded:

Some relatives … asked how[transport] had gone afterwards and in a way that they may not have before. (Participant 5)

Relatives signify the patient's real-world relationships, and when they describe how [the patients] doing and that they have children and grandchildren, you immediately get emotional. (Participant 4)

### Post-Transport and Reflections

#### Decontaminating helicopters post-transport

After transporting a patient with COVID-19, the helicopter received an extensive, thorough cleaning that included spraying stretchers and other equipment with antiseptics. Participants described how the need for decontamination and the careful handling of waste complicated transport. Early in the pandemic, helicopters were decontaminated by spraying down everything; more recently, however, decontamination has been performed more easily with a hydrogen peroxide machine, which has also afforded more space for equipment during transport. Helicopters cleaned with the machine are thus ready for new assignments immediately after delivery. Also at the start of the pandemic, participants had to fly home wearing their PPE and then sanitise the helicopter. They described how restoring the equipment after transporting patients with COVID-19 meant that the helicopter was locked for an extended period:

We couldn't use the new alarms until we finished decontamination, … and once we were done, we had to contact SOS again to say that we were ready for new assignments. (Participant 2)

How do you clean [a helicopter]? Everything that it requires takes an incredibly long time. (Participant 6)

#### Impacting one's private life

Some participants described that working with patients with COVID-19 did not affect their private lives, while others described putting their own agendas on hold. They reported that preparing for transport had often required them to work overtime in 2020 and that the care staff's quality of life had deteriorated as a consequence. Unable to exercise or sleep regularly, they were usually exhausted but also wanted to avoid becoming infected at work and spreading the virus to their families at home. They thus maintained distance from their families and would not hug their children for fear of spreading the virus. For many, once they had finished working, it took a full day before they dared to live normally again:

You continually realised that working with many COVID patients during a shift made you a slight risk, so you felt, at least initially, that you needed to keep your distance to wait and see whether you’d been infected. (Participant 5)

It's stress overload. If you get infected, then you’ll take it home to your family and children. To avoid that, you ideally want to move into the garage and be away from home, but that rarely works, because everyone has a life. It's not 2 weeks we’re talking about; we’ve been doing this for a hell of a year. … There are many who have suffered incredibly in their personal lives. (Participant 3)

#### Gaining new experiences after working with patients with COVID-19

Participants described resolving situations in the best way possible based on the conditions at hand. They emphasised learning how to adjust to the unprecedented and described now knowing more about COVID-19 in ways that will make countering the next pandemic easier. In that light, their experience was excellent training for the future, and they indeed stressed the need to be prepared for such situations:

We’ve probably all learned and adjusted to something that we—that no one—was prepared for. Not even people who should’ve been prepared for [the pandemic] were prepared. (Participant 1)

To expand the helicopter operation, the participants proposed establishing warehouses with the most up-to-date equipment and recruiting more care staff as needed. More than anything, participants underscored the importance of having a good care team whose members can support each other.

What I’ve learned above all is how important it is to have a good group and to be able to help each other. (Participant 4)

## Discussion

Our findings demonstrate that the participating nurses constantly needed to stay updated as routines and guidelines were revised. Similar results have emerged in other studies whose participants struggled to keep abreast with the large influx of information provided to them (Goh et al., 2020; Nowell et al., 2021; Schroeder et al., 2020). In particular, Nowell et al. (2021) found that excessive or irrelevant information had caused their participants stress and, in response, advised establishing a contact person to provide regular updates regarding changes in resources and guidelines.

Although the nurses in our study described fearing PPE shortages, they ultimately always had access to such equipment. In Robinson and Stinson’s (2021) study, care staff also feared shortages, and in most other studies, shortages in fact occurred (Ardebili et al., 2021; Catania et al., 2021; Sun et al., 2020; Villar et al., 2021), or else the equipment provided was of poor quality (Catania et al., 2021; Villar et al., 2021). Such trends indicate that care staff's access to PPE during the pandemic has differed, which, from an intersectional perspective, means that the conditions for good work environments have differed depending on the location.

The nurses described working in PPE as being hot and numbing. In other studies, care staff have similarly expressed feeling overheated while working in PPE (Galehdar et al., 2020a, 2020b; Jose et al., 2021; Villar et al., 2021). Facial pain due to wearing protective face masks was also commonly described by our participants, which corroborates past findings of similar pain and pressure injuries due to PPE (Jose et al., 2021; Villar et al., 2021). Because several studies have described discomfort associated with working in PPE (Ardebili et al., 2021; Galehdar et al., 2020a; Gordon et al., 2021; Jose et al., 2021; Sun et al., 2020), healthcare professionals should be afforded access to individually tailored PPE in order to avoid pain while working with patients with infectious diseases. More broadly, additional research regarding PPE is required to afford care staff working environments with good conditions for recovery.

Due to PPE, participants also felt distanced from patients and emphasised the difficulty of achieving nurse–patient intimacy. In other studies, nurses have described how PPE prevented them from touching patients (Silverman et al., 2021) and erected a barrier to care (Robinson & Stinson, 2021). In Fernández-Castillo et al.’s (2021) study, reduced contact and a lack of closeness with patients caused by PPE emerged as factors that impaired nursing. According to Joyce Travelbee's relationship theory, nurse–patient interaction is pivotal for patients to receive good care, and to meet patients’ needs with empathy, nurses should view patients as people with similar feelings (Shelton, 2016). Getting to know patients is as important as performing care, for it improves the likelihood of detecting not only obvious changes but also subtle ones in patients’ diseased states. Travelbee's theory maintains that patients should be approached as unique individuals and that care should be person-centred. In our study, patient–nurse interaction may have been negatively affected by the nurses’ having to work in PPE. More research regarding patients’ experiences with being cared for by staff with PPE is therefore required to optimise care for patients.

Our results show that along with the background noise in helicopters, respiratory protection impaired communication during helicopter transport. That finding corroborates the results of Sandström et al. (2017) and Senften and Engström (2015), who found that communication was limited due to the background noise in helicopters. Goh et al. (2020) have also described difficulties with communicating due to PPE. Our participants reported communicating with physicians by using body language (e.g. eye contact and hand gestures) or by writing on paper or a board when they could not hear each other, which aligns with the use of non-verbal communication in helicopter transport under normal circumstances found in other research (Senften & Engström, 2015). Thus, communication via body language occurs not only when working in PPE. Although it remains unclear how communication under those conditions affects patients’ safety, a fundamental part of Travelbee's theory is that good communication is necessary for good nursing (Shelton, 2016).

Our participants additionally expressed fearing the virus's high contagiousness, which reflects results from past studies on working with COVID-19 (Arcadi et al., 2021; Fernández-Castillo et al., 2021; Gunawan et al., 2021; Kackin et al., 2020; Moradi et al., 2021). Our participants also described feeling insecure about not having cared for patients with COVID-19 before. Justifying their concerns, Galehdar et al. (2020b) and Silverman et al. (2021) have revealed shortcomings in knowledge about treating patients with COVID-19, such that patients’ suffering from respiratory problems that nurses could not remedy negatively impacted the nurses (Galehdar et al., 2020b). Kellogg et al. (2021) have thus highlighted the need for personal and institutional support for nurses to cope with distress from providing care during the pandemic.

Our participants added that the care staff's quality of life had deteriorated and that they were exhausted due to lack of sleep and regular exercise. That finding confirms results from González-Gil et al.’s (2021) study, in which nursing staff felt emotionally drained at the end of the workday and had trouble sleeping. In the research of Kackin et al. (2020) and Moradi et al. (2021), participants experienced symptoms in the form of anxiety and depression after work during the pandemic. Our participants highlighted the benefit of having a care team whose members could support each other, which confirms past studies whose participants reported receiving similar support from their own care teams (Arcadi et al., 2021; Goh et al., 2020). To improve the quality of life of each care staff member, the employer should provide individually tailored support.

Our participants’ fear of being infected at work and spreading the virus at home has also emerged in other studies (Ardebili et al., 2021; Catania et al., 2021; Galehdar et al., 2020a, 2020b; Goh et al., 2020; González-Gil et al., 2021; Iheduru-Anderson, 2021; Kackin et al., 2020; Moradi et al., 2021; Robinson & Stinson, 2021; Schroeder et al., 2020; Villar et al., 2021). Our participants reported maintaining distance from their families after working with patients with COVID-19, and other studies have shown similar results, namely that nurses avoided close contact with their families after working with patients with COVID-19 (Arcadi et al., 2021; Ardebili et al., 2021; Catania et al., 2021; Galehdar et al., 2020a, 2020b). They additionally described waiting a full day after working with such patients before daring to live normally again. That finding is consistent with past results showing that care staff did not want to meet their family members for several days for fear of spreading the virus (Galehdar et al., 2020b). Our participants also kept their distance from their children and avoided hugging them, which Galehdar et al. (2020b) found could induce anxiety and stress for nurses who are parents. More recently, Schierberl Scherr et al. (2021) highlighted the importance of social support as a protective factor against depressive symptoms amongst nurses working with patients with COVID-19 and the need to be close to family in such situations.

### Strengths and Limitations

Our study's results have limitations. For example, only six specialist nurses were interviewed, which is a rather small sample. Even so, the interviews were rich in content, and the nurses described similar experiences, which suggested a pattern that served as an adequate basis for our findings (Brinkmann & Kvale, 2018). Moreover, the participants, setting, data collection and analysis have been described thoroughly to enable readers to evaluate the findings’ transferability to other contexts but while also ensuring the participants’ confidentiality. As we are also specialist nurses in intensive care nursing and nursing researchers with clinical ICU experience both before and during the COVID-19 pandemic, our understanding might have discouraged us from asking follow-up questions. However, we viewed our preunderstanding as a resource and remained aware of the importance of asking such questions.

## Implications for Practice

Feelings of distance between patients and nurses in connection with helicopter transport need to be reduced, especially when using PPE. Ensuring the adequacy of equipment and information required in the event of major accidents and pandemics is critical, as is ensuring that staff members are safe in relation to the equipment and medications used and not at risk of infection.

## Conclusion

Unlike intensive care helicopter transport for patients without COVID-19 or without other contagious infections, such transport for patients with COVID-19 aims to prevent the spread of infection. Despite the participating nurses’ anxiety over potential PPE shortages, their fear subsided once they realised that the PPE was effective. Working in PPE nevertheless created a feeling of distance from patients, compromised nurse–patient intimacy, and adversely affected communication between nurses, patients, physicians, and pilots. Working with patients with COVID-19 additionally affected the participants’ private lives by keeping them at a distance from their families in order to lower the risk of infection. The regular testing of the care staff for COVID-19, if desired, could have counteracted those suspicions and given the staff better conditions for recovery. On the plus side, our participants felt that they could depend on their care teams for support. In view of those results, more research on patients’ experiences with being cared for by staff wearing PPE is needed, as is more general research on working with infectious diseases while wearing PPE. Such research can ensure better work environments for healthcare professionals during future pandemics, not least in special healthcare environments such as helicopters, and in improving the situation for healthcare professionals could also improve the situation for patients.
